# Late-Onset Euglycemic Diabetic Ketoacidosis in a Patient With Massive Stroke Requiring Decompressive Craniectomy: A Case Report

**DOI:** 10.7759/cureus.18629

**Published:** 2021-10-09

**Authors:** Syed Ahmed Hussaini, Afia Aziz, Muzamil Musa, Mohammed Alamin, Mohammed Danjuma

**Affiliations:** 1 Psychiatry, Hamad Medical Corporation, Doha, QAT; 2 Internal Medicine, Hamad Medical Corporation, Doha, QAT

**Keywords:** dm, euglycemic dka, type 2 diabetes mellitus, stroke, dapagliflozin, sglt-2 inhibitors, diabetic ketoacidosis

## Abstract

Euglycemic diabetic ketoacidosis (DKA) is a well-recognized adverse effect associated with the use of sodium-glucose co-transporter-2 (SGLT-2) inhibitors. Early recognition of this medical emergency and timely intervention can prevent the notorious consequences of this serious complication. However, this form of DKA can easily be masqueraded by normal serum glucose levels. This article describes a 49-year-old man diagnosed with type 2 diabetes mellitus (DM) on dapagliflozin who presented with a large right-sided middle cerebral artery (MCA) stroke complicated by euglycemic DKA, developed 72 hours after stopping the drug. This case is unique considering that dapagliflozin's half-life is only 12.9 hours, and the body completely eliminates it within 72 hours. But our patient developed DKA features after the elimination window. Hence, this case highlights the importance to consider euglycemic DKA in the presence of ketonemia and metabolic acidosis in a patient who is a chronic SGLT-2 inhibitor user even if the drug was discontinued several days before the patient's presentation.

## Introduction

Euglycemic diabetic ketoacidosis (DKA) is an unusual form of DKA that presents with a high anion gap metabolic acidosis, increased ketonemia, and a normal glucose level, which at the time of presentation makes it a diagnostic dilemma [1]. Early recognition of this medical emergency and timely intervention can prevent fatal consequences like electrolyte imbalances, cerebral and pulmonary edema. SGLT-2 inhibitors are FDA-approved drugs that help lower blood glucose by reducing the reabsorption of glucose in proximal convoluted tubules of the kidney, leading to a low insulin-to-glucagon ratio because high blood glucose levels are the primary stimulus for insulin release. However, one of the severe adverse effects of SGLT-2 inhibitors is euglycemic DKA, which is increasingly being reported due to the growing demand for SGLT-2 inhibitors [2].

Factors like prolonged fasting, major illnesses, intense exercise, excessive alcohol intake, or an abrupt reduction in concurrent insulin dose can trigger euglycemic DKA in patients taking SGLT-2 inhibitors. These conditions can aggravate relative insulin deficiency, to the point of stimulating ketogenesis [2].

This article discusses a 49-year-old man with a past medical history of type 2 diabetes mellitus (DM), metabolic syndrome, and current drug history of dapagliflozin, presenting with a large right-sided middle cerebral artery (MCA) stroke, complicated by euglycemic DKA 72 hours after drug discontinuation. Despite his normal serum glucose levels upon presentation, he was found to have ketonemia and high anion gap metabolic acidosis consistent with DKA.

This case is peculiar with its unusual presentation. It gives physicians a chance to consider the diagnosis of late-onset euglycemic DKA in patients who may present with high anion gap metabolic acidosis and normal glucose levels in high-stress situations like stroke despite the withdrawal of SGLT-2 inhibitors.

## Case presentation

A 49-year-old male, known to have DM for 15 years, hypertension, coronary artery disease, and a previous episode of left parietal stroke three months back (had no residual weakness and was on follow-up for previous stroke) presented to the Emergency Department (ED) with sudden onset left-sided body weakness and headache. These symptoms developed six hours prior to presentation when he woke up at 8 a.m and noticed a left-sided facial droop. He denied any other neurological symptoms. His antidiabetic medications were sitagliptin/metformin HCL 1000/50 mg 2 tabs daily, gliclazide 120 mg daily, and dapagliflozin 10 mg daily (for the last five years). Other daily medications included valsartan, bisoprolol, clopidogrel, isosorbide dinitrate, trimetazidine, atorvastatin, and rabeprazole.

The patient's blood pressure was 135/80 mmHg, while the other vitals were within normal limits. The patient's Glasgow Coma Scale was 12/15. Neurological examination on presentation was significant for complete left-sided hemiplegia, left hemisensory loss, left facial weakness (upper motor neuron lesion), and positive Babinski's sign bilaterally. Cerebellar and other cranial nerves examination was unremarkable. There were no signs of meningeal irritation. Examination otherwise was normal.

**Figure 1:**
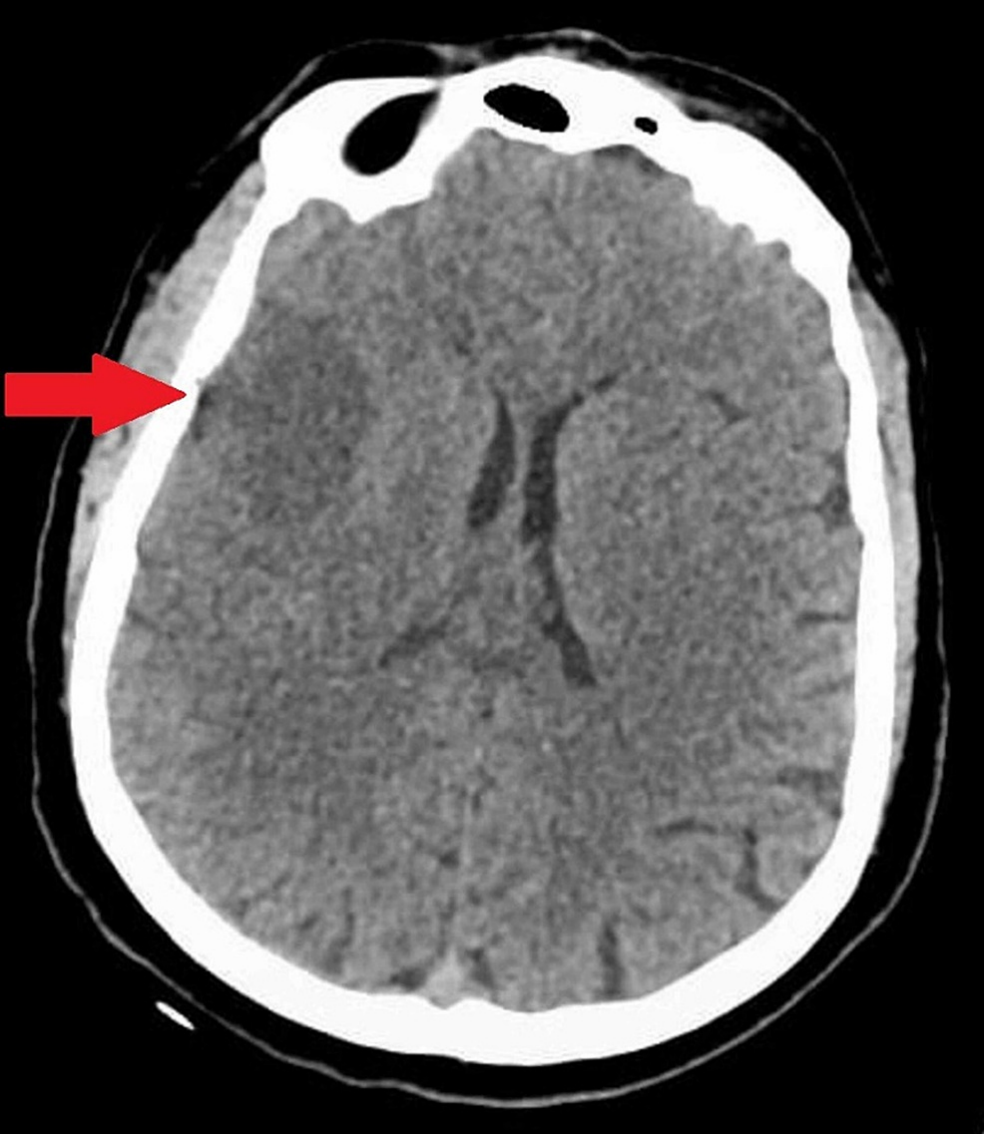
Initial non-contrast CT head on the day of admission. The arrow indicates the ischemic region that is visible as a hypodense fronto-parietal area extending to the insular cortex and external capsule. CT: Computed Tomography

Initial plain computed tomography (CT) scan head showed a right frontal, cortical, and subcortical hypodense area extending to the insular cortex and external capsule consistent with acute ischemic infarct as shown in Figure 1.

The patient did not undergo thrombolysis at that time as he presented beyond the thrombolysis window and was managed conservatively. Relevant laboratory evaluation on admission is mentioned in Table 1.

**Table 1:** Lab findings on admission. INR: international normalized ratio; HCO3: bicarbonate; HDL: high-density lipoprotein; LDL: low-density lipoprotein

Laboratory Tests	Admission	Normal Range
WBC Count	9.3 ×10^9^/L	4–10 ×10^9^/L
Hemoglobin (Hb)	14.6 gm/dL	13-17 gm/dL
Platelets	191 ×10^3^/µL	150-400×10^3^/µL
INR	1.1	<1.5
Serum Glucose	9.1 mmol/L	3.3-5.5 mmol/L
Urea	3.9 mmol/L	2.8-8.1 mmol/L
Creatinine	71 umol/L	62-106 umol/L
Sodium	138 mmol/L	136-145 mmol/L
Potassium	4.2 mmol/L	3.5-5.1 mmol/L
HCO3	23 mmol/L	22-29 mmol/L
Serum Albumin	37 gm/L	35-52 gm/L
Serum Cholesterol	2.7 mmol/L	< 5.2 mmol/L
Serum Triglycerides	0.7 mmol/L	< 1.7 mmol/L
Serum HDL	0.7 mmo/L	> 1.0 mmo/L
Serum LDL	1.7 mmol/L	<3.36 mmol/L

The patient was kept in a fasting state for the next 24 hours and received intravenous (I/V) fluids only. Enteral feed was started later through a nasogastric tube. Oral hypoglycemic agents were discontinued on presentation, and blood glucose was managed via insulin aspart sliding scale.

On day three of hospitalization, the patient became somnolent and difficult to arouse. While arranging the CT head of this patient to look for progression of stroke and possible re-bleed, other causes were also sought especially as his routine labs showed bicarbonate levels to be trending downwards from 23 mmol/L at the time of admission to 12 mmol/L. Arterial blood gas (ABG) analysis showed high anion gap metabolic acidosis with normoglycemia. His renal function tests and lactate levels were normal, excluding uremia and lactic acidosis as possible causes. Testing for DKA with ketone levels had not been performed because of normal blood glucose concentrations. While keeping in mind this patient's long-time use of SGLT-2 inhibitors prior to hospital admission, we initiated screening for DKA by measuring β-Hydroxybutyrate levels, which were elevated. These labs are shown in Table 2.

**Table 2:** Lab findings on day 3 and day 12 of admission. pCO2: partial pressure of carbon dioxide

Lab Parameters	Day 3	Day 12
Arterial pH (7.35-7.45 mmHg)	7.168	7.53
Blood Urea Nitrogen (2.8-8.1 mmol/L)	4.1	4.7
Serum Creatinine (62-106 µmol/L)	91	54
Serum Sodium (132-146 mmol/L)	137	141
Serum Potassium (3.5-5.1 mmol/L)	4.6	3.3
Serum Chloride (98-107 mmol/L)	106	105
Serum Bicarbonate (22-29 mmol/L)	12	25
Serum Glucose (3.3-5.5 mmol/L)	8.9	6.5
Serum Lactate (0.5-2.2 mmol/L)	1.6	-
pCO2 (35-45 mmHg)	37	27
Anion Gap (6-12 mmol/L)	20.3	12.3
Serum β -hydroxybutyrate (0.03-0.3 mmol/L)	4.1	0.63

**Figure 2:**
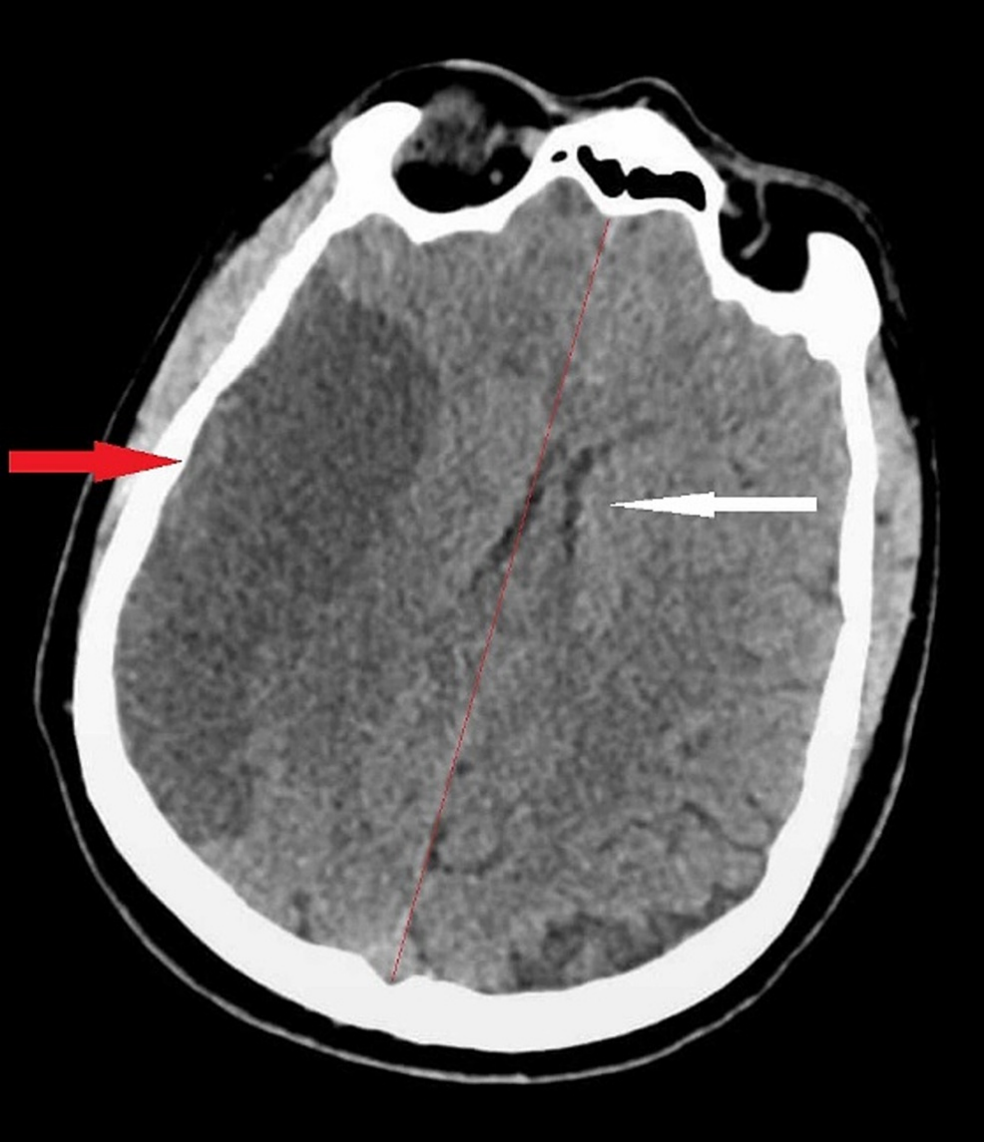
Non-contrast CT head on day 3. Red arrow depicts the ischemic area extending through fronto-parietal and temporal areas. White arrow shows mass effect in the form of effacement of the adjacent cortical sulci and ipsilateral lateral ventricle with midline shift towards the left side of 5.5 mm CT: Computed Tomography

**Figure 3:**
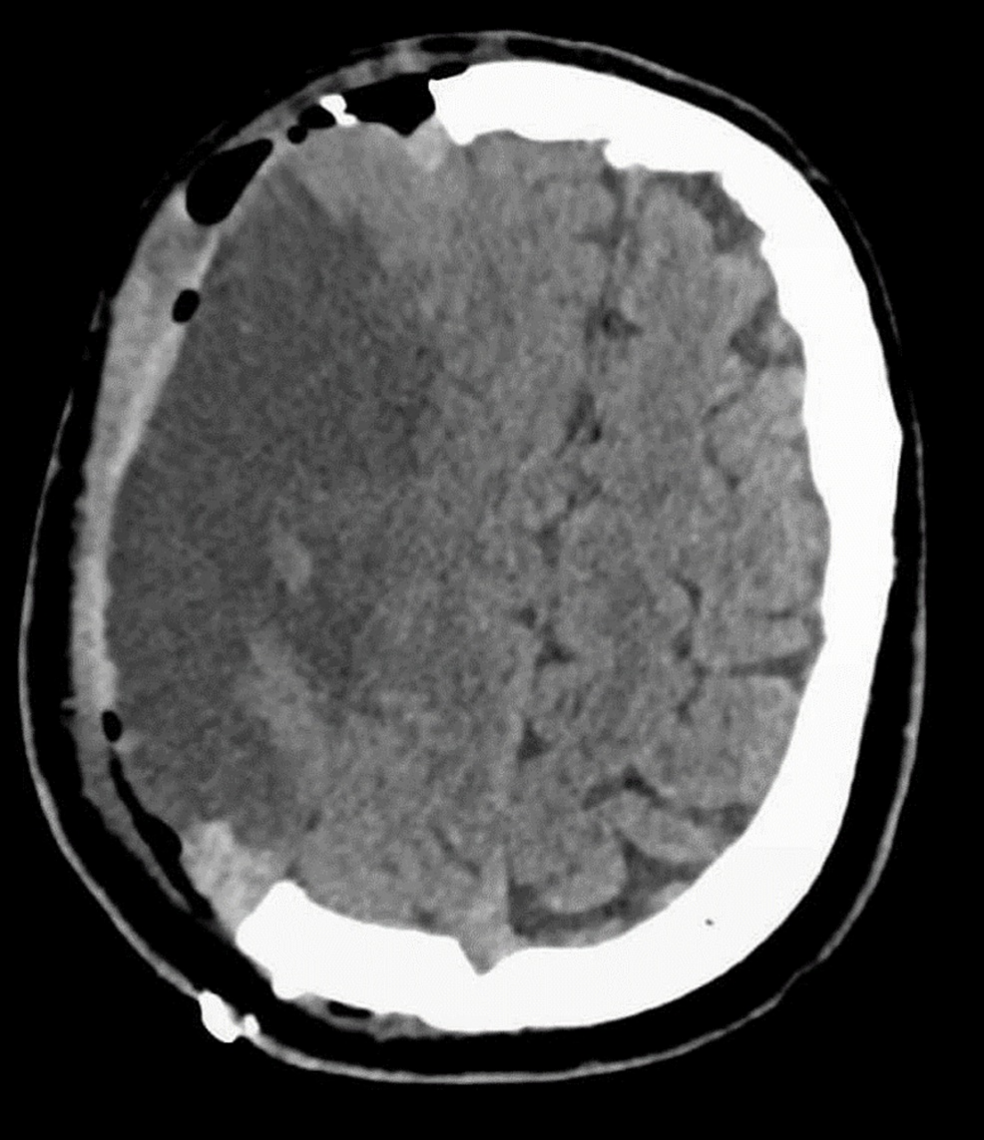
Non-contrast CT head showing postoperative changes at the right hemicranium in the form of right frontoparietal craniectomy and extra-axial hematoma CT: Computed Tomography

The patient was also tested for anti-GAD antibodies in order to look for an element of type 1 diabetes mellitus (LADA), but he tested negative. As the patient was taking dapagliflozin for his DM management for five years, which was stopped on admission, a diagnosis of euglycemic DKA related to SGLT2 inhibitor therapy was made. The patient was treated with intravenous fluids, dextrose, and insulin according to local and international guidelines for DKA management. Serial blood gas analyses showed gradual resolution of his ketoacidosis. Unfortunately, due to the severity of brain injury resulting from his large stroke, the patient's clinical condition worsened. CT head repeated on the same day when DKA developed revealed a midline shift and extension of infarction site for which he underwent immediate decompressive craniectomy as shown in Figures 2, 3.

After the operation, he was transferred to the surgical intensive care unit (SICU) for continuity of care with further optimization of his diabetic ketoacidosis management done in the SICU. The patient remained in SICU for almost one month, after which he was shifted to a rehabilitation facility with multidisciplinary team care. He was discharged after a prolonged and complicated stay in the hospital for six months and is currently placed on follow-up after one month with the rehabilitation department.

## Discussion

Euglycemic diabetic ketoacidosis (DKA) has always been a diagnostic challenge for physicians due to its insidious presentation and requires a high clinical suspicion. In order to diagnose euglycemic DKA, three conditions need to be fulfilled: the presence of high anion gap metabolic acidosis, positive urinary or serum ketones, and normoglycemia that is blood glucose less than 200 mg/dl or 11.1 mmol/L [3,4]. With 101 case reports published of euglycemic DKA secondary to SGLT-2 inhibitor use, our case is unique as it is the second reported case of late-onset euglycemic DKA after three days of discontinuation of dapagliflozin [5].

Dapagliflozin has a half-life of 12.9 hours [6]. It should be eliminated completely from the body after five to six half-lives, approximately within three days. Our patient developed euglycemic DKA after the drug has been stopped for around 72 hours; this late presentation has been reported only once in literature in an article by Iqbal et al. [5]. This might be explained by the fact that the drug can stay in the body for a considerable amount of time beyond three days and continue to exert its pharmacological effects. So clinicians should be aware that even if SGLT-2 inhibitors have been stopped for some time, they are still capable of exerting their pharmacological effects.

In addition to euglycemic DKA, other adverse effects associated with SGLT-2 inhibitors include mycotic genital infections as well as an increased risk of acute kidney injury, venous thromboembolism, bone fracture, and acute pancreatitis [7].

Common aggravating factors of euglycemic DKA include inappropriate insulin reduction or omission among those who were insulin-deficient, bariatric surgery, excessive alcohol intake, physical exertion, and dietary restriction (low-carbohydrate or reduced intake) [8]. Our patient had multiple factors that could predispose him to euglycemic DKA, including reduced dietary intake secondary to dysphagia as a consequence of massive right-sided middle cerebral artery (MCA) stroke as well as prolonged SGLT-2 inhibitor (dapagliflozin), and insulin secretagogue (gliclazide) use. Both of them were stopped at admission and the patient was placed on insulin.

Apart from SGLT-2 inhibitor use, there are other causes that have been reported in the literature to precipitate euglycemic DKA, including prolonged fasting, insulin use prior to hospital admission, pregnancy, cocaine abuse, pancreatitis, and liver cirrhosis [4]. However, SGLT-2 inhibitors have recently gained interest among physicians because the mechanisms by which they cause euglycemic DKA still remain poorly understood, although several mechanisms have been proposed.

The exact pathophysiology of how SGLT-2 inhibitors cause euglycemic DKA is unknown, but it is thought that decreased serum glucose due to glycosuric effects of SGLT-2 inhibitors, and reduced carbohydrate intake, leads to a decrease in insulin secretion. Low insulin levels, in turn, lead to increased glucagon secretion, which further leads to accelerated lipolysis releasing free fatty acids that undergo oxidation to produce keto acids and thus causing ketosis. Furthermore, lipolysis is augmented in diabetics who are already in a state of insulin deficiency. A second proposed mechanism of euglycemic DKA is thought to be due to SGLT-2 inhibitors increasing reabsorption of ketones by the kidneys resulting in an increase in serum ketone levels. This renal effect of lipolysis and ketone reabsorption by SGLT-2 inhibitors is thought to mimic starvation-induced ketosis. In short, glucosuria and ketosis in the setting of insulin deficiency can lead to euglycemic DKA by SGLT-2 inhibitor use [9,10].

Despite the increased number of euglycemic DKA episodes in the past few years, this drug class is gaining fair popularity due to its tremendous advantages, which not only includes regulation of glucose levels but also extends to the improvement of lipid profile, and reducing blood pressure and albuminuria [11]. An additional advantage is its off-label use in type 1 diabetics, although it is still not FDA approved but gaining considerable evidence of benefit in this patient population, who already have limited medication regime to be treated with [12]. In fact, ipragliflozin, an SGLT-2 inhibitor currently available in Japan, Korea, and Thailand, is now approved in Japan for use along with insulin in adults with type 1 diabetes [13]. With respect to advantages in type 2 DM patients, marked improvement in cardiovascular and renal outcomes has been observed, as well as a decline in in-patient hospitalization secondary to heart failure even in patients without diabetes [14].

So, despite the side effect profile, the advantages merit its continuous use in medicine, and its use will continue to trend upwards as it is incorporated for use in diabetic patients with micro and macro- complications. Therefore, it is of immense importance to study these side-effects in detail, to enhance the clinical awareness of physicians and the ability to identify such events when they occur and be vigilant in the management of these patients.

## Conclusions

Euglycemic DKA has become a rising complication in patients stabilized on SGLT-2 inhibitors. Late-onset euglycemic DKA is a cause of increased morbidity and calls for extra vigilance on the part of clinicians involved in the management of these patients especially in acute settings. Patients on these drugs should also be counseled and educated about the signs and symptoms of DKA despite normal blood glucose levels. Moreover, caution should be taken while administrating SGLT-2 inhibitors in patients with multiple comorbidities and risk factors. Hence, an awareness of the side effect profile of these drugs while taking into account the patient's comorbidities and judicious use of them may prevent avoidable complications.
